# Papulonecrotic Tuberculid

**DOI:** 10.4269/ajtmh.17-0377

**Published:** 2017-10-11

**Authors:** Valeti Meghana, Gowtham Saravanan, Kaliaperumal Karthikeyan

**Affiliations:** Department of Dermatology, Venereology and Leprosy, Sri Manakula Vinayagar Medical College and Hospital, Puducherry, India

**Figure 1.**
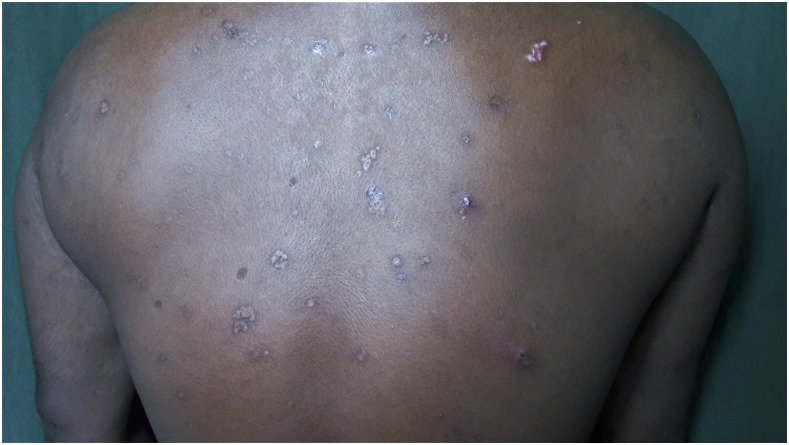
Multiple, hyperpigmented, crusted papules with varioliform scarring seen over the back.

**Figure 2.**
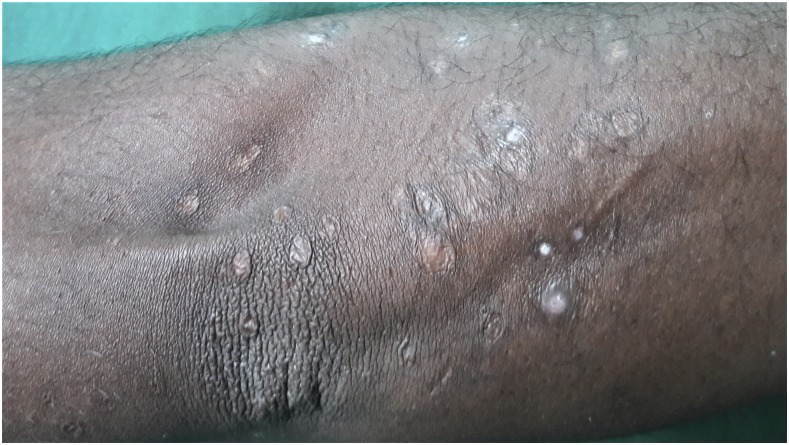
Atrophic varioliform scarring over extensor aspect of left forearm.

**Figure 3.**
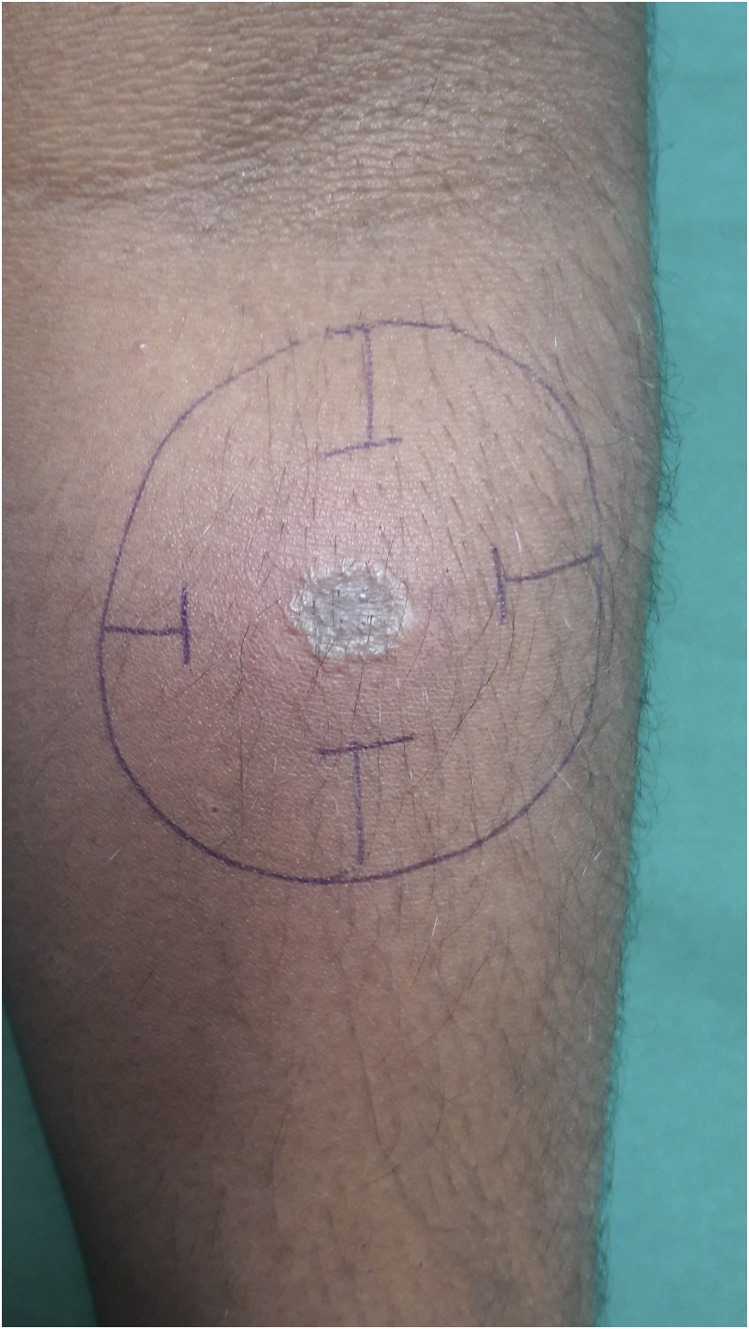
Positive Mantoux reaction (23 × 23).

**Figure 4.**
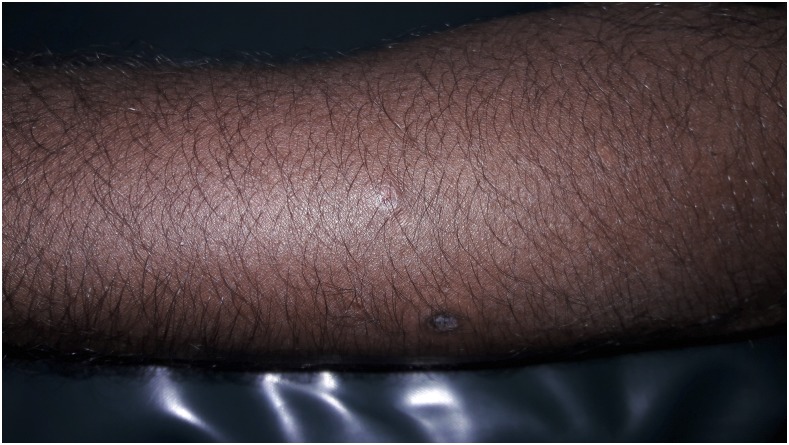
Single, crusted papule from which skin biopsy was taken.

A 30-year-old man presented with a 6 year history of recurrent, multiple asymptomatic raised lesions over his back and bilateral upper limbs. He had been treated repeatedly for a case of recurrent boils with oral and topical antibiotics. Some of the lesions had healed spontaneously leaving behind unsightly scars. The patient denied any history of associated fever, chronic cough, weight loss, and drug intake prior to the onset of lesions. There was no recognized contact with tuberculosis patients. General and systemic examination was essentially normal. Dermatological examination revealed the presence of multiple, well-defined, hyperpigmented crusted papules of 0.5–1.0 cm in size, distributed symmetrically over his entire back, extensor surface of bilateral forearm, arm, and bilateral dorsum of foot, interspersed with atrophic varioliform scarring (Figures 1 and 2). Routine laboratory workup was normal. Tuberculin (Mantoux) was strongly positive at 72 hours (23 × 23 mm) with central necrosis (Figure 3). Sputum for acid fast bacilli culture, chest radiograph, ultrasound abdomen, and pelvis did not reveal any abnormality. A biopsy specimen taken from a crusted papule over his forearm (Figure 4) showed fibrinoid necrosis, surrounded by mixed inflammatory infiltrate filling the entire dermis along with a few ill-defined epitheloid granulomas and lymphocytoclastic vasculitis. These are consistent with papulonecrotic tuberculid.

The patient was started on a four drug combination therapy of rifampicin, isoniazid, pyrazinamide, and ethambutol for two months initially followed by a combination of rifampicin and isoniazid to complete a total of 6 months of standard antitubercular therapy. The patient responded well and all of the lesions eventually healed.

Tuberculids are hypersensitivity reactions to *Mycobacterium tuberculosis* or its products in individuals with good immunity.^1^ Papulonecrotic tuberculid is a relatively uncommon manifestation of cutaneous tuberculosis.^2^ It presents as chronic, recurrent, symmetrical eruption of necrotizing papules that ulcerate, crust, and heal after a few weeks with varioliform scarring.^3^ Hallmarks of this disease include positive mantoux test, evidence of present or past tuberculosis, inability to isolate *M. tuberculosis* in the skin lesions, resolution of lesions with atrophic varioliform lesions, and response to antitubercular therapy.^1^

In a country like India where the prevalence of tuberculosis is high, existence of papulonecrotic tuberculid is possible and the focus of the infection may not be demonstrable in the majority of cases. Once diagnosed patients respond very well to antituberculosis therapy. It is important to consider papulonecrotic tuberculid as a differential diagnosis in any patient with recurrent eruption that heals with scarring in an endemic area.
